# HIV and Aging: Tracking the Epidemic Via the 30-year History of GSA’s HIV Interest Group

**DOI:** 10.1093/geroni/igaa057.3110

**Published:** 2020-12-16

**Authors:** Charles Emlet

## Abstract

The HIV and Aging Interest Group at GSA begin30 years ago. At that time, Highly Active Antiretroviral Treatments (HAART) did not exist. The Ryan White CARE act was newly enacted and older adults living with HIV/AIDS were primarily infected in later life. HIV was considered a life threatening disease. This Interest Group‘s existence has paralleled the epidemic including its challenges, innovations and rewards. The number of sessions focusing on HIV and aging has grown at GSA, with shifting emphasis toward longevity, quality of life and comorbidity. This symposium, celebrating the theme of “Turning75: Why age Matters” calls upon past and current IG conveners, including the founding convener, to review the history of the HIV and Aging interest group. Tepper discusses the group’s origins and the historical context of HIV as an emerging issue for older people. Emlet will discuss the changes in HIV care and treatment during the early2000s and how these advances were reflected in the interest group and GSA sessions. Brennan-Ing reviews significant changes between2012 through2015, when the proportion of older adults with HIV was projected to surpass50%, and the NIH Office of AIDS Working Group on HIV and Aging published its first set of recommendations. Finally, Taylor and colleagues brings us to the IG’s current activities including the importance of HIV Cohort studies and national policy highlights such as the National HIV/AIDS and Aging Awareness Day. The symposium provides a thoughtful account of the importance of this interest group. HIV, AIDS and Older Adults Interest Group Sponsored Symposium.

